# Isolated Port-Site Recurrence after Laparoscopic Distal Pancreatectomy for Pancreatic Adenosquamous Carcinoma: A Case Report

**DOI:** 10.70352/scrj.cr.26-0347

**Published:** 2026-09-11

**Authors:** Satoshi Nishiwada, Tetsuya Tanaka, Masato Takano, Yuki Kirihataya, Takeshi Takei, Tomomi Sadamitsu, Hiroki Nakahara, Hazuki Horiuchi, Atsushi Yoshimura, Tadashi Nakagawa

**Affiliations:** 1 Department of Surgery, Minami-Nara General Medical Center, Yoshino, Nara, Japan; 2 Department of Pathology, Minami-Nara General Medical Center, Yoshino, Nara, Japan; 3 Department of Gastroenterology, Minami-Nara General Medical Center, Yoshino, Nara, Japan

**Keywords:** port-site recurrence, laparoscopic distal pancreatectomy, pancreatic adenosquamous carcinoma

## Abstract

**INTRODUCTION:**

Minimally invasive pancreatectomy has become widely adopted worldwide. However, its oncologic safety in malignant diseases remains a matter of ongoing debate, including atypical patterns of tumor recurrence. Port-site recurrence (PSR) is a rare but clinically relevant condition following minimally invasive oncologic surgery. Although several mechanisms, including tumor manipulation and pneumoperitoneum-related factors, have been proposed, the underlying pathogenesis remains unclear. Especially in pancreatic cancer, reports of PSR after minimally invasive surgery are extremely limited, and its optimal management has not been established.

**CASE PRESENTATION:**

A 75-year-old man was referred to our institution with a pancreatic tail tumor detected on CT during evaluation of epigastric pain. Contrast-enhanced CT and endoscopic US-guided fine-needle aspiration biopsy led to a diagnosis of pancreatic cancer, and laparoscopic distal pancreatectomy was performed as upfront surgery. Intraoperatively, no peritoneal dissemination was observed, and peritoneal cytology was negative. The pancreatic tail tumor demonstrated invasion of the anterior pancreatic serosa. Histopathological examination revealed adenosquamous carcinoma composed of approximately 70% squamous cell carcinoma and 30% adenocarcinoma components. The postoperative course was uneventful, and adjuvant chemotherapy with S-1 was administered. Approximately 6 months after surgery, the patient developed a painful mass at the left lower trocar site. Contrast-enhanced CT and abdominal ultrasonography revealed a 3 × 4-cm irregular mass within the rectus abdominis muscle at the corresponding trocar site, with no evidence of distant metastasis. The lesion was considered an isolated PSR based on multidisciplinary discussion, and surgical resection was performed. Intraoperative findings confirmed the absence of peritoneal dissemination, and the tumor was completely resected. Pathological examination revealed metastatic squamous cell carcinoma, consistent with the squamous component of the primary tumor, leading to a diagnosis of PSR. The patient remains free of recurrence 6 months after resection.

**CONCLUSIONS:**

PSR after minimally invasive surgery for pancreatic cancer is extremely rare. In this case, tumor biology, especially the squamous component in adenosquamous carcinoma, may play a key role in its development. Surgical resection may be considered in carefully selected patients with isolated PSR, although further studies are required to clarify its clinical significance.

## Abbreviations

CA19-9
: carbohydrate antigen 19-9
CEA
: carcinoembryonic antigen
DUPAN-2
: Duke pancreatic monoclonal antigen type 2
LDP
: laparoscopic distal pancreatectomy
PSR
: port-site recurrence
TNM
: tumor–node–metastasis
UICC
: Union for International Cancer Control

## INTRODUCTION

With the rapid advancement of surgical technology, minimally invasive surgery has become widely adopted worldwide. Minimally invasive pancreatectomy, in particular for benign and low-grade pancreatic tumors, has gained increasing acceptance as a feasible alternative to open surgery, supported by international consensus guidelines and RCTs demonstrating its safety and feasibility.^1–3)^ However, its oncologic safety in malignant diseases remains a matter of ongoing debate, including atypical patterns of tumor recurrence. Recent RCTs have demonstrated comparable recurrence-free survival between laparoscopic and open distal pancreatectomy without demonstrating clear oncologic superiority.^1,4)^

PSR is a rare but potentially serious complication following laparoscopic surgery and is thought to be related to tumor dissemination associated with pneumoperitoneum and surgical manipulation.^5–8)^ Although PSR has been described in various malignancies, its occurrence after laparoscopic pancreatectomy for pancreatic cancer is exceedingly limited.^9,10)^ Herein, we present a case of PSR after LDP for pancreatic adenosquamous carcinoma and discuss the possible mechanisms underlying this rare event.

## CASE PRESENTATION

**Fig. 1:**
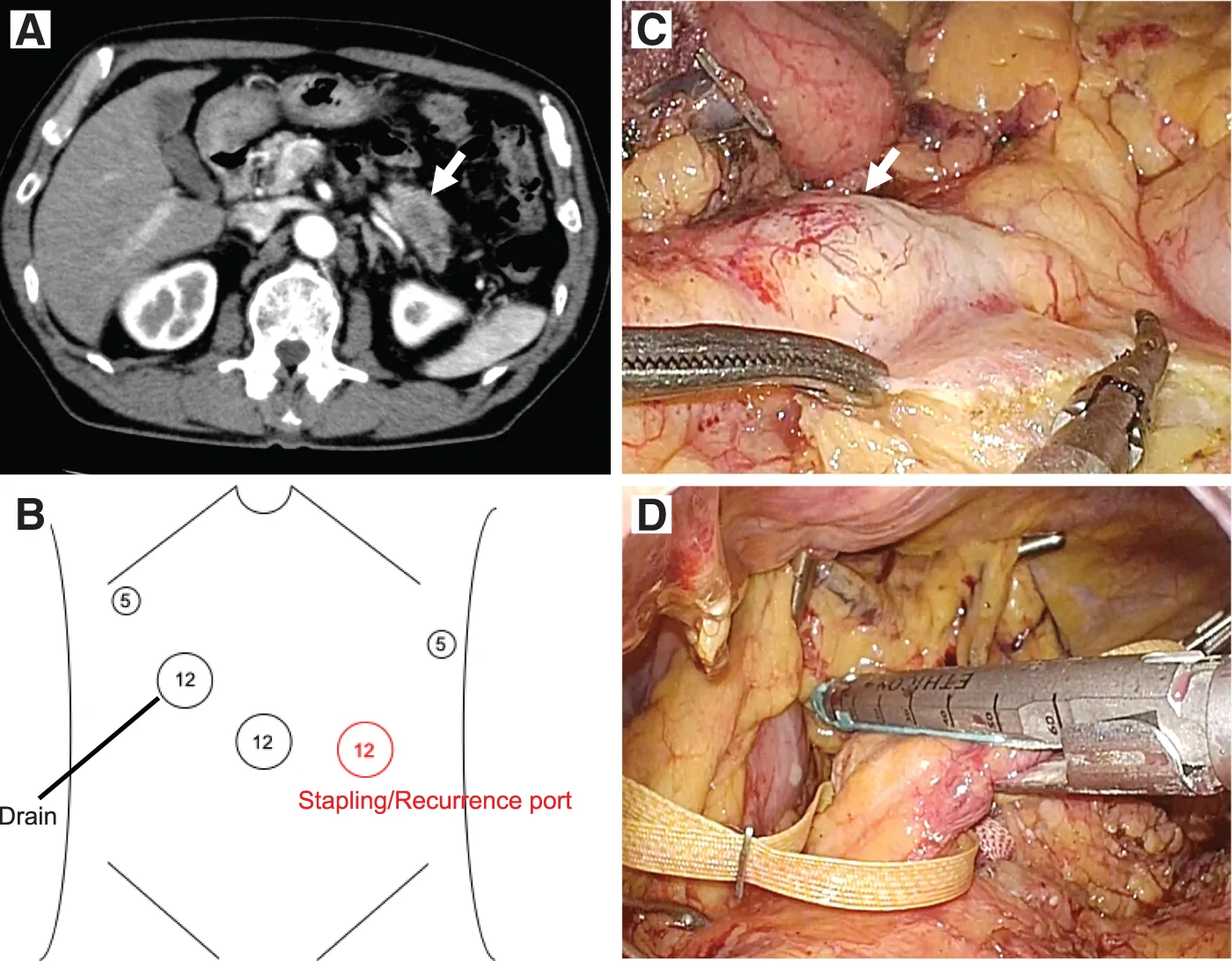
CT image and intraoperative findings of the pancreatic tail tumor. (**A**) Contrast-enhanced CT showed a low-density lesion approximately 25 mm in size in the pancreatic tail (arrow). (**B**) Schematic illustrations of port placement. (**C**) The pancreatic tail tumor demonstrated invasion of the anterior pancreatic serosa (arrow). (**D**) Pancreatic transection was performed using a linear stapler inserted through a 12-mm trocar placed in the left lower abdomen.

A 75-year-old man on medical treatment for vasospastic angina and gastroesophageal reflux disease presented with epigastric pain that had persisted for approximately 3 months. A CT performed for further evaluation revealed a suspected tumor in the pancreatic tail, and the patient was referred to our institution. Contrast-enhanced CT at our institution demonstrated a low-density lesion approximately 25 mm in size in the pancreatic tail without any distant metastases (**Fig. 1A**). Endoscopic US-guided fine-needle aspiration confirmed the diagnosis of pancreatic tail cancer, suspected adenosquamous carcinoma, and the patient was subsequently referred to our surgical department. As the patient declined neoadjuvant therapy, upfront LDP was planned.

**Fig. 2:**
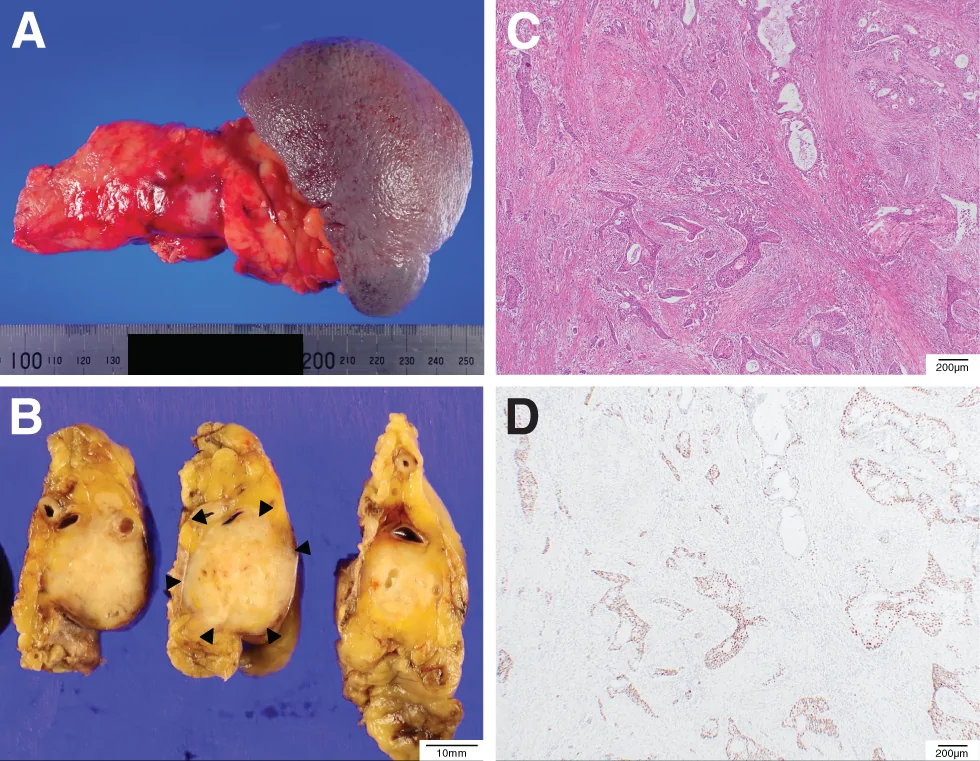
Gross appearance and pathological findings of the resected pancreatic tumor. (**A**, **B**) Gross examination of the resected specimen revealed a yellowish-white mass measuring approximately 20 × 19 × 13 mm in the pancreatic tail (black arrowheads). (**C**, **D**) Histopathological examination showed that the tumor consisted of approximately 70% squamous cell carcinoma components and 30% adenocarcinoma components, leading to a diagnosis of adenosquamous carcinoma (**C**: hematoxylin and eosin staining; **D**: immunohistochemical staining for p40).

The operation was carried out using a 5-port laparoscopic approach (**Fig. 1B**). During surgery, we confirmed the absence of peritoneal dissemination and negative peritoneal lavage cytology. The pancreatic tail tumor demonstrated invasion of the anterior pancreatic serosa, with partial involvement of the transverse mesocolon (**Fig. 1C**). Pancreatic transection was performed using a linear stapler inserted through a 12-mm trocar placed in the left lower abdomen, with a gross transection margin of approximately 5 cm from the tumor (**Fig. 1B** and **1D**). Intraoperative frozen-section analysis confirmed a negative pancreatic transection margin. The resected specimen was retrieved through the umbilical port site using a protective retrieval bag after extending the umbilical incision to approximately 4 cm. The postoperative course was uneventful, with no clinically relevant postoperative pancreatic fistula or other complications, and the patient was discharged on POD 10. Gross examination of the resected specimen revealed a yellowish-white mass measuring approximately 20 × 19 × 13 mm in the pancreatic tail, and the distance between the primary tumor and the pancreatic transection margin was 55 mm (**Fig. 2A** and **2B**). Histopathological examination showed that the tumor consisted of approximately 70% squamous cell carcinoma and 30% adenocarcinoma components, leading to a diagnosis of adenosquamous carcinoma with no lymph node metastasis (**Fig. 2C** and **2D**). The tumor was classified as pT1cN0M0, Stage IA according to the UICC TNM classification (8th edition), with negative resection margins (R0). Adjuvant chemotherapy with S-1 was subsequently administered.

**Fig. 3:**
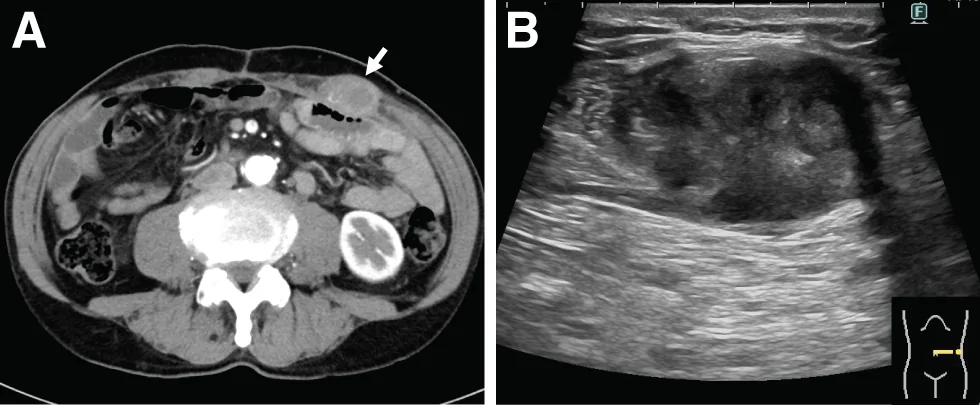
CT and ultrasonography findings of the PSR lesion. (**A**) Contrast-enhanced CT and (**B**) abdominal ultrasonography revealed a 3 × 4-cm irregular mass within the rectus abdominis muscle at the corresponding trocar site (white arrow). PSR, port-site recurrence

**Fig. 4:**
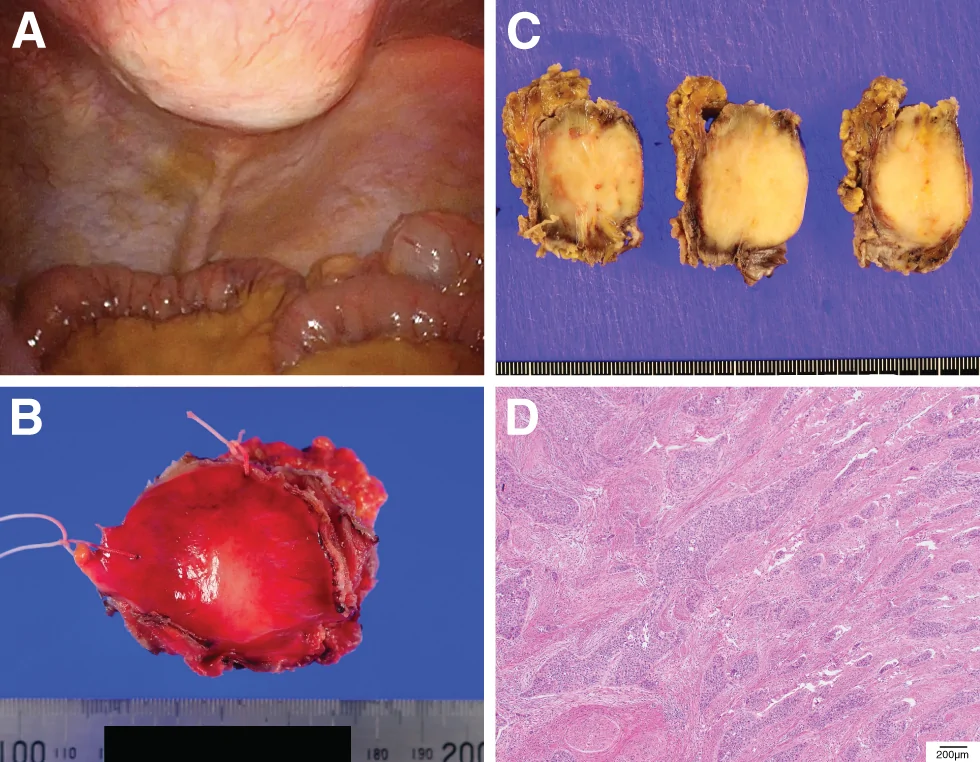
Intraoperative and histopathological findings of the PSR lesion. (**A**) Laparoscopic examination revealed that the port-site recurrent lesion appeared as a well-defined, elevated lesion with a slightly whitish appearance, covered by the peritoneum. (**B**, **C**) The surgically resected specimen grossly presented a yellowish-white mass measuring approximately 30 × 25 × 25 mm, similar to the previously resected pancreatic tumor. (**D**) Histopathological examination showed predominantly squamous cell carcinoma with a minimal adenocarcinoma component, resembling the squamous component of the primary pancreatic tumor (hematoxylin and eosin staining). PSR, port-site recurrence

Approximately 6 months after surgery, the patient presented with induration and pain at the left lower abdominal port site (**Fig. 1B**). Contrast-enhanced CT and abdominal ultrasonography revealed a 3 × 4-cm irregular mass within the rectus abdominis muscle at the corresponding trocar site, with no evidence of distant metastasis (**Fig. 3A** and **3B**). Following multidisciplinary discussion at our gastroenterological tumor board, the lesion was considered an isolated PSR without evidence of distant metastasis. Laboratory investigations revealed no significant abnormalities, and serum CA19-9, CEA, and DUPAN-2 levels remained within the normal range throughout the clinical course, including at the time of initial surgery and at the diagnosis of PSR (**Table 1**). Given the possibility of complete resection, the patient’s significant local pain, and his favorable performance status, surgical resection was selected. Diagnostic laparoscopy confirmed the absence of distant and peritoneal metastases, and peritoneal cytology was negative. Laparoscopic examination revealed that the port-site recurrent tumor appeared as a well-defined, elevated lesion with a slightly whitish appearance, covered by the peritoneum (**Fig. 4A**). A 5-cm skin incision was made over the tumor, and full-thickness resection of the abdominal wall, including the rectus abdominis muscle and peritoneum, was performed. Primary closure of the muscular layer was achieved. The patient recovered without any postoperative complications and was discharged on POD 7.

**Table 1:** Laboratory findings at the PSR diagnosis

Hematology	
WBC	6600/μL
RBC	384 × 10^4^/μL
Hemoglobin	14.8 g/dL
Platelet	15.1 × 10^4^/μL
Blood chemistry	
CRP	0.16 mg/dL
T-Bil	0.76 mg/dL
D-Bil	0.22 mg/dL
AST	20 U/L
ALT	19 U/L
ALP	157 U/L
γ-GTP	27 U/L
LDH	156 U/L
ChE	227 U/L
CK	75 U/L
AMY	51 U/L
TP	6.7 g/dL
Albumin	3.9 g/dL
Prealbumin	19.1 mg/dL
Uric acid	3.38 mg/dL
BUN	15.7 mg/dL
Creatinine	0.82 mg/dL
TC	148 mg/dL
TG	118 mg/dL
Na	135.2 mEq/L
K	4.52 mEq/L
Cl	98.6 mEq/L
Ca	9.51 mg/dL
Tumor markers	
CEA	3.02 ng/mL
CA19-9	18.2 U/mL
DUPAN-2	<25 U/mL

ALP, alkaline phosphatase; ALT, alanine aminotransferase; AMY, amylase; AST, aspartate aminotransferase; BUN, blood urea nitrogen; CA19-9, carbohydrate antigen 19-9; CEA, carcinoembryonic antigen; ChE, cholinesterase; CK, creatine kinase; CRP, C-reactive protein; D-Bil, direct bilirubin; DUPAN-2, Duke pancreatic monoclonal antigen type 2; LDH, lactate dehydrogenase; PSR, port-site recurrence; RBC, red blood cell; T-Bil, total bilirubin; TC, total cholesterol; TG, triglyceride; TP, total protein; WBC, white blood cell; γ-GTP, γ-glutamyltranspeptidase

The surgically resected specimen grossly presented a yellowish-white mass measuring approximately 30 × 25 × 25 mm within the rectus abdominis muscle layer, similar to the previously resected pancreatic tumor (**Fig. 4B** and **4C**). Histopathological examination showed almost exclusively squamous cell carcinoma, resembling the squamous component of the primary pancreatic tumor (**Fig. 4D**). Based on the clinical course, imaging findings, and histopathological findings, we diagnosed the lesion as PSR of pancreatic cancer. The surgical margins were negative. Serum squamous cell carcinoma antigen was measured after resection of the PSR, following the pathological diagnosis, and was also within the normal range (1.0 ng/mL). The patient remains alive without evidence of recurrence 6 months after resection of the PSR lesion.

## DISCUSSION

The advent of minimally invasive surgery has led to significant advancements and innovations in surgical practice. PSR is a rare but clinically relevant complication following minimally invasive oncologic surgery; however, its underlying mechanisms remain to be fully elucidated.^5–8)^ Moreover, only a limited number of cases of PSR after minimally invasive surgery for pancreatic cancer have been reported.^9,10)^ Several possible mechanisms of port-site metastasis have been proposed, including tumor manipulation, tumor exposure, aerosolization of tumor cells under pneumoperitoneum, the chimney effect caused by gas leakage, the favorable microenvironment of healing wounds, and specimen retrieval without protection.^5,8,11)^ These mechanisms are considered to contribute to tumor cell dissemination and implantation at trocar sites. However, these factors alone cannot fully explain the occurrence of port-site metastasis, and tumor-related factors, particularly tumor aggressiveness, are also considered to play a crucial role.^8,11,12)^ In the present case, the PSR lesion was located primarily within the rectus abdominis muscle with focal invasion into the adjacent peritoneum. This anatomical distribution appears less consistent with secondary involvement of the abdominal wall from peritoneal dissemination and may suggest direct implantation of tumor cells into the abdominal wall during surgical manipulation. However, the precise mechanism of PSR remains speculative.

In the present case, the primary pancreatic tumor was an adenosquamous carcinoma, a relatively rare histological subtype known for its aggressive biological behavior and poorer prognosis compared with adenocarcinoma. To the best of our knowledge, PSR of pancreatic adenosquamous carcinoma has not been previously reported. Given that tumor aggressiveness has been reported as a potential factor associated with the development of PSR, the aggressive biological behavior of this tumor subtype, adenosquamous carcinoma, may have contributed to tumor cell dissemination and implantation in the port site. An additional interesting finding in the present case is the difference in histological composition between the primary tumor and the PSR. While the primary tumor consisted of approximately 70% squamous cell carcinoma and 30% adenocarcinoma components, the port-site recurrent lesion had almost exclusively a squamous cell carcinoma component. This novel finding may suggest that the squamous component in pancreatic cancer possesses a higher malignant potential and plays a dominant role in tumor dissemination and implantation, leading to the development of PSR.

In terms of surgical factors, intraoperative findings revealed invasion of the anterior pancreatic serosa, which could have increased the likelihood of tumor cell exposure during surgical manipulation. We did not identify any operative factors clearly associated with the development of PSR. Previous reports have suggested that port-site metastasis may occur at sites subjected to instrument use or tumor handling.^8,11)^ In pancreatic cancer, PSR at a previous drain insertion site has been reported; however, a causal relationship between drain placement and PSR remains unclear.^9,10)^ In the present case, the surgical drain was placed through a different port site (**Fig. 1B**). Interestingly, the PSR developed at a trocar site used during pancreatic transection with a surgical stapler. Although a direct causal relationship has not been established, it is possible that local tumor cell contamination during surgical manipulation contributed to tumor implantation at this site. Further accumulation of cases across multiple institutions is warranted to clarify the relationship between surgical technique and PSR. However, because the proposed tumor implantation hypothesis and its association with surgical manipulation remain unproven, alternative mechanisms, including hematogenous metastasis, cannot be excluded.

Because the underlying mechanisms of PSR remain poorly understood and the condition is extremely rare, the optimal treatment management of PSR in pancreatic cancer remains unclear. Before considering surgical resection, careful evaluation for systemic disease is essential, as PSR may represent a manifestation of disseminated tumor spread rather than a purely localized recurrence. In the present case, contrast-enhanced CT and abdominal ultrasonography revealed no evidence of distant metastases. Although ^18^F-fluorodeoxyglucose PET/CT was not performed, it might have provided additional information for detecting occult metastatic disease. However, diagnostic laparoscopy and negative peritoneal cytology confirmed the absence of peritoneal dissemination and supported the diagnosis of isolated PSR. Alternative approaches, such as systemic chemotherapy with close radiological surveillance or radiotherapy for local control and symptom palliation, might also have been considered, although evidence supporting these approaches is currently limited. In the present case, surgical resection was selected based on the following considerations: (1) the lesion was considered an isolated recurrence without evidence of distant metastasis and was therefore potentially amenable to curative resection; (2) the patient experienced significant local pain and preferred surgical treatment; and (3) the patient maintained a favorable performance status and was considered fit for surgery. We carefully explained to the patient that the oncologic benefit of surgical resection for PSR has not been established and that evidence supporting this approach remains limited. After obtaining informed consent, surgical resection was performed. To date, it has been reported that surgical resection was performed for PSR of pancreatic cancer in only 2 cases.^9,10)^ Among these, resection for isolated PSR without other metastatic lesions has been described in only 1 case reported by Aida et al., in which the patient achieved recurrence-free survival for 15 months after resection for PSR.^9)^ In contrast, Park et al. reviewed 8 cases of PSR at their institution and reported that surgical resection for PSR was performed in only 1 patient with concomitant peritoneal metastasis, who subsequently died of pancreatic cancer 8 months after surgery.^10)^ In the present case, although the postoperative observation period is limited to 6 months, the patient has remained recurrence-free following resection of an isolated port-site lesion. While further accumulation of cases and long-term follow-up are essential to establish the oncologic benefit of surgical resection for PSR, surgical resection may be considered a treatment option in carefully selected patients, especially those with solitary PSR without evidence of other distant metastases.

## CONCLUSIONS

We have reported a case of PSR following LDP for pancreatic adenosquamous carcinoma. This study suggests that tumor aggressiveness, particularly the squamous component in this case, may play a key role in the development of PSR. Further accumulation of evidence is required to clarify the underlying mechanisms and optimal treatment management of this rare but clinically significant condition.

## DECLARATIONS

### Availability of data and materials

The dataset supporting the conclusions of this article is available in the manuscript.
